# Synthetic Receptors Induce Anti Angiogenic and Stress Signaling on Human First Trimester Cytotrophoblast Cells

**DOI:** 10.3390/ijerph14050517

**Published:** 2017-05-11

**Authors:** Ahmed F. Pantho, Mason Price, AHM Zuberi Ashraf, Umaima Wajid, Maryam Emami Khansari, Afsana Jahan, Syeda H. Afroze, Md Mhahabubur Rhaman, Corey R. Johnson, Thomas J. Kuehl, Md. Alamgir Hossain, Mohammad Nasir Uddin

**Affiliations:** 1 Department of Biochemistry, University of Texas at Austin, Austin, TX 78712, USA; ahmedfaiaz1995@utexas.edu; 2 Department of Biology, Angelo State University, San Angelo, TX 76904, USA; mprice15@angelo.edu; 3 Department of Obstetrics & Gynecology, Baylor Scott & White Health, Temple/Texas A&M Health Science Center College of Medicine, Temple, TX 76508, USA; xeonflow16@gmail.com (A.Z.A.); Umaima.Wajid@BSWHealth.org (U.W.); Thomas.Kuehl@BSWHealth.org (T.J.K.); 4 Department of Chemistry & Biochemistry, Jackson State University, Jackson, MS 39217, USA; mary.emami@yahoo.com (M.E.K.); afsanajahan01@gmail.com (A.J.); md_mhahabubur.rhaman@jsums.edu (M.M.R.); corey19144@gmail.com (C.R.J.); 5 Medical Physiology, Texas A&M Health Science Center College of Medicine, Temple, TX 76504, USA; Afroze@medicine.tamhsc.edu; 6 Department of Pediatrics, Baylor Scott & White Health, Temple/Texas A&M Health Science Center College of Medicine, Temple, TX 76508, USA; TKUEHL@sw.org; 7 Internal Medicine, Baylor Scott & White Health, Temple/Texas A&M Health Science Center College of Medicine, Temple, TX 76508, USA

**Keywords:** angiogenic, cardiotonic steroids, cell signaling, cytotrophoblast, preeclampsia, synthetic receptors

## Abstract

The cytotrophoblast (CTB) cells of the human placenta have membrane receptors that bind certain cardiotonic steroids (CTS) found in blood plasma. One of these, marinobufagenin, is a key factor in the etiology of preeclampsia. Herein, we used synthetic receptors (SR) to study their effectiveness on the angiogenic profile of human first trimester CTB cells. The humanextravillous CTB cells (Sw.71) used in this study were derived from first trimester chorionic villus tissue. Culture media of CTB cells treated with ≥1 nM SR level revealed sFlt-1 (Soluble fms-like tyrosine kinase-1) was significantly increased while VEGF (vascular endothelial growth factor) was significantly decreased in the culture media (* *p* < 0.05 for each) The AT_2_ receptor (Angiotensin II receptor type 2) expression was significantly upregulated in ≥1 nM SR-treated CTB cells as compared to basal; however, the AT_1_ (Angiotensin II receptor, type 1) and VEGFR-1 (vascular endothelial growth factor receptor 1) receptor expression was significantly downregulated (* *p* < 0.05 for each). Our results show that the anti-proliferative and anti-angiogenic effects of SR on CTB cells are similar to the effects of CTS. The observed anti angiogenic activity of SR on CTB cells demonstrates that the functionalized-urea/thiourea molecules may be useful as potent inhibitors to prevent CTS-induced impairment of CTB cells.

## 1. Introduction

Preeclampsia (preE) [1] is a hypertensive disorder of pregnancy characterized by hypertension (diastolic ≥90 mmHg) and proteinuria (≥300 mg in 24 h) after 20 weeks of gestation. PreE occurs in 5–10% of pregnancies [2]. PreE is the second leading cause of maternal and fetal morbidity and mortality in the world [3,4,5,6]. The incidence of preE has risen in the U.S. [7,8]. PreE is often accompanied by intrauterine growth restriction (IUGR) and is associated with preterm birth. Both of the latter conditions prejudice the survival and well being of the fetus [9]. There is no definitive therapy other than delivery.

Cardiotonic steroids (CTS) [10,11] are endogenous steroidal inhibitors of the Na^+^/K^+^ ATPase (NKA). Vasoconstrictor digitalis-like CTS are a second natriuretic system. In addition to vaso-relaxant atrial natriuretic peptides [12], CTS are key factors in the regulation of blood pressure. A number of CTS have been identified in human plasma and urine, including cardenolides (OUB) (e.g., endogenous ouabain (EO) and digoxin) and bufadienolides (e.g., marinobufagenin (MBG), bufalin, and resibufogenin). Binding to the CTS receptor site on the α-subunit of the NKA induces natriuresis [3,13,14]. In addition to transport of Na^+^ and K^+^, the NKA functions as a receptor, which is capable of transducing CTS binding into activation of intracellular protein kinases and alterations in Ca^2+^ levels, ultimately altering the cell-surface expression of the NKA and Na^+^/H^+^ exchanger [15]. In this light, CTS can be viewed as a new class of hormones, rather than simply as endogenous inhibitors of Na^+^ transport. Recent data support a role for CTS, specifically MBG, in the pathogenesis of some forms of hypertension, including preE [3,4,16]. Among the numerous factors contributing to the development of preE is the significant role played by the CTS and the imbalance of angiogenic and antiangiogenic factors [17]. There are two types of CTS: OUB and bufadienolides [MBG and Cinobufotalin (CINO)]. MBG may be secreted in response to the stimulus of excessive volume expansion in the first trimester in sensitive pregnant patients. It has been shown that urinary MBG levels are elevated prior to the development of hypertension, indicating that it may play a key role in the pathogenesis of preE. CTS are stimulated to promote natriuresis via inhibition of the Na^+^/K^+^ pump in renal tubules and are likely to exhibit a prohypertensiveaction via inhibition of the Na^+^/K^+^ pump in vascular sarcolemma. CTS inhibit Na^+^/K^+^ ATPase in vascular smooth muscle cells; the elevation of local Na^+^ facilitates Ca^2+^ entry through NCX1, resulting in vasoconstriction [18].

MBG levels are elevated in preE. PreE is a syndrome which is generally believed to involve multiple etiologic factors. Current research focuses mainly on three pathogenetic mechanisms; (1) over-secretion of MBG, (2) an imbalance of pro- and anti-angiogenic factors, and (3) agonistic autoantibodies to the angiotensin II type 1 receptor [3]. Recent data strongly support the involvement of MBG in preE. CTS ‘endogenous digoxin-like factors’ (EDLF) [11] have been known since the 1980s to increase significantly during pregnancy-induced hypertension and preE [19,20,21]. Recently, MBG was identified as the EDLF. MBG, but not EO, is markedly elevated in PE [22]. Puschett and co-workers developed an ELISA with high specificity for MBG [23], which revealed a five-fold increase in serum MBG and a four-fold increase in urine MBG levels in preeclamptic patients versus normotensive pregnant women [24]. Plasma levels of MBG, but not EO, become elevated in patients with moderate PE [25]. In addition to the effects of MBG on Na^+^K^+^ ATPase and fibrotic signaling, MBG has also been shown to interfere with the proliferation, migration, and invasion of cytotrophoblast (CTB) cells [26,27,28] and to disrupt endothelial cell junctions [29,30]. Recently, we demonstrated that CINO impedes Sw.71 CTB cell line function via cell cycle arrest and apoptotic signaling [31].

Disruption of angiogenic balance by MBG is hypothesized to contribute to preE [32,33,34]. Angiogenic imbalance during pregnancy may contribute to preE [35,36,37]. When administered to pregnant rats, endogenous sFlt-1 and sEng induce several features of preE, including hypertension, proteinuria, and HELLP syndrome [38]. PlGF is expressed in the placenta and is proangiogenic [39]. sFlt-1 inhibits PlGF. In preE patients, both sFlt-1 and sEng levels are increased prior to clinical symptoms and also correlate with the severity of the disease, whereas PlGF levels are significantly decreased [40]. The sFlt-1/PlGF ratio, while not yet FDA approved, appears to have diagnostic and predictive value in patients at risk of developing PE [40], and tests like the CobasElecsys^®^ (Rotkreuz, Switzerland) for preE (sFlt-1 & PlGF) (Roche) are increasingly used in the clinical management of PE [41]. MBG has been shown to decrease expression of pro-angiogenic factors VEGF and PlGF, while up-regulating expression of anti-angiogenic factors sFlt-1 and sEng in a cell line of extravillous CTBs. These effects were seen between 0.1 nM and 1 nM for MBG, which is within the physiological range 0.25 + 0.02 nM [22]. Antibodies to CTS decrease the blood pressure in preE. Additional evidence for the role of CTS in preE comes from studies in which intravenously administered Digibind lowered blood pressure in patients with PE [20,21]. It has been suggested that MBG induces vascular fibrosis via a Fli-1-dependent mechanism, thus suggesting that MBG represents a potential target for therapy of preE [42].

**Figure 1:**
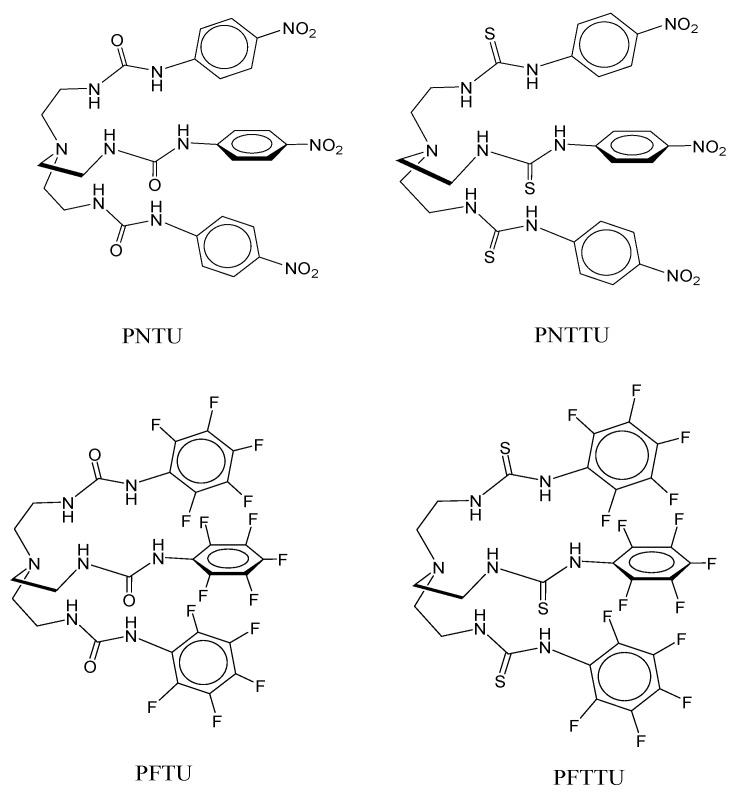
Chemical structures of acyclic receptors: PNTU (Para Nitro Tripodal Urea), PFTU (Penta Fluoro Tripodal Urea), PNTTU (Para Nitro Tripodal Thio-Urea), and PFTTU (Penta Fluoro Tripodal Thio-Urea).

**Figure 2:**
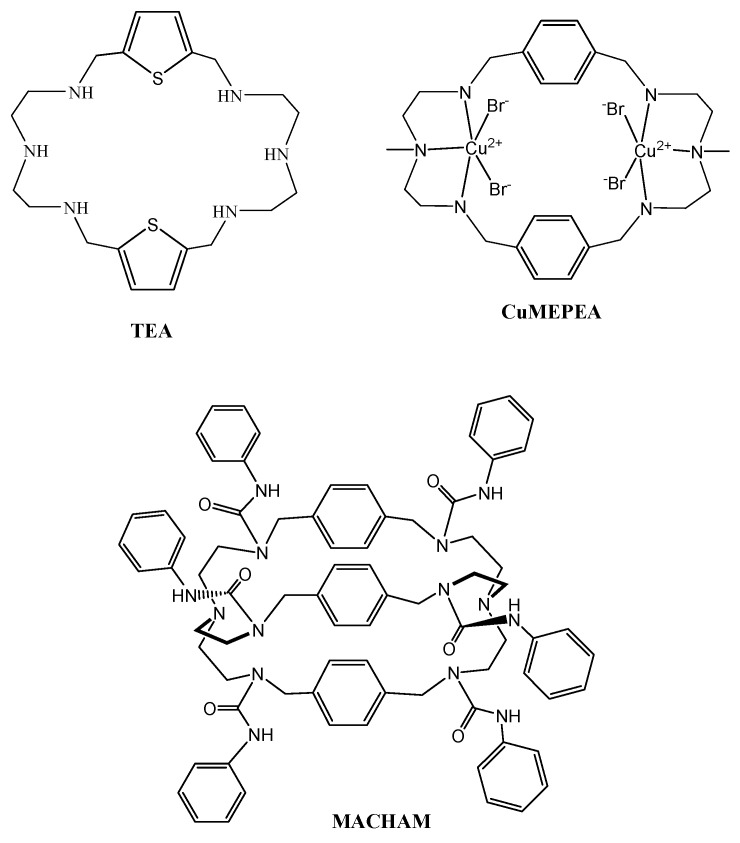
Chemical structures of cyclic receptors: TEA (Thiophene-based Ethylene Macrocyclic Amine), CuMEPEA (Copper(II) complex of Methylated Para-xylyl-based Ethylene Macrocyclic Amine) and MACHAM (Macrocyclic Amide).

Synthetic receptors (SR) are architecturally designed by chemists for selective recognition of a variety of substrates, mimicking many critical enzymatic activities involved in biology [43,44,45,46,47,48,49,50,51]. The unique functional groups present in such molecules can be potentially used for designing drugs to target specific activities in a human body [45]. Recently, we used a synthetic receptor on CTB cells, demonstrating that it impaired the function of human first trimester CTB cells [52]. In order to better understand the cell surface receptors for CTS and their interactions with synthetic receptors, we studied four acyclic compounds (Figure 1): Para Nitro Tripodal Urea (PNTU), Penta Fluoro Tripodal Urea (PFTU), Para Nitro Tripodal Thio-Urea (PNTTU), and Penta Fluoro Tripodal Thio-Urea (PFTTU) and three cyclic compounds (Figure 2): Thiophene-based Ethylene Macrocyclic Amine (TEA), Copper(II) complex of Methylated Para-xylyl-based Ethylene Macrocyclic Amine (CuMEPEA), and Macrocyclic Amide (MACHAM). As shown in Figure 1, the basic feature of these compounds is that each of them contains three active functional groups as urea [HN(C=O)NH] groups (PNTU and PFTU) or thiourea [HN(C=S)NH] groups (PNTTU and PFTTU) in the attached three arms forming a triopdal cavity. In addition, the presence of electron-withdrawing groups on the aromatic units enhances the acidity of the attached NH groups, thereby increasing the overall activity of these molecules. In addition, the conformational flexibility with six H-donor groups may allow them to interact with cells. In order to understand the effectiveness of such compounds, we included three macrocyclic-based compounds, as shown in Figure 2, featuring amine (TEA), metal center (CuMEPEA), and amide (MACHAM) functional groups.

While preE is considered to be a multifactorial disease, a consensus among preE researchers has emerged that MBG levels are important in at least some cases [32]. MBG levels are elevated in most PE patients. In vitro data demonstrate the effects of MBG on endothelial function [30] and cell growth [53] and, importantly, on proliferative and angiogenic signalling in CTB cells [26,27,28]. Compelling data from multiple animal models, in which MBG levels are elevated prior to the onset of preE, suggests that MBG may represent a relevant target for preE therapeutics. Indeed, the administration of DigiBind was effective at alleviating hypertension in preE patients. Anti-MBG antibodies have alleviated preE symptoms in a rodent model. We have identified a novel anti-MBG human monoclonal antibody, which binds with high affinity to NKA and attenuates MBG-induced anti-proliferative and anti-angiogenic signaling in a CTB cell model [34], as an innovative, effective, and safe therapeutic for preE.

In the present study, we investigated the seven selected synthetic receptors with different function groups to explore their effectiveness on the angiogenic profile of human first trimester CTB cells.

## 2. Materials and Methods

### 2.1. General Information

The chemicals used for this work were purchased from Sigma-Aldrich as reagent grades and were used as received. Nuclear magnetic resonance (NMR) spectra were recorded at 25 °C on a Varian Unity INOVA 500 FT-NMR. Chemical shifts for NMR were expressed in parts per million (ppm) and calibrated against trimethylsilane (TMS) or sodium salt of 3-(trimethylsilyl)propionic-2,2,3,3-*d*_4_ acid (TSP) as an external reference used in a sealed capillary tube. All NMR data were processed and analyzed with MestReNova Version 6.1.1-6384 (Mestrelab, Escondido, CA, USA). Mass spectral data were obtained at ESI-MS positive mode on a FINNIGAN LCQDUO (Jackson State University, Jackson, MS, USA). Elemental analysis was done from Columbia Analytical Service (Tucson, AZ, USA). The CTB cell culture media DMEM/F-12 was purchased from Invitrogen, Grand Island, NY, USA, and the cells were incubated in an Isotemp CO_2_ Incubator, Fisher, Waltham, MA, USA. RPMI Media and gels were purchased from Invitrogen, Grand Island, NY, USA. Cell viability and cell proliferation assay kits were purchased from Promega, Madison, WI, USA. A cell migration assay kit was purchased from Cell Biolabs, San Diego, CA, USA. The BCA protein assay kit and chemiluminescent substrate were from Pierce, Rockford, IL, USA. The Quantikine ELISA was purchased from R&D Systems, Minneapolis, MN, USA. The nitrocellulose membranes were from Bio-Rad, Hercules, CA, USA. The primary and secondary antibodies were purchased from Santa Cruz Biotechnology, Paso Robles, CA, USA; Abcam, Cambridge, MA, USA and Jackson Immuno Research Laboratories, West Grove, PA, USA. Absorbance was measured on a plate reader, SPECTRA max 340PC384, Molecular Devices, Sunnyvale, CA, USA. The fluorescence was measured on a fluorescence plate reader, CytoFluor Series 4000 Fluorescence Multi-Well Plate Reader, Applied Biosystems, Grand Island, NY, USA. The chemiluminence detection system used was the LAS-3000 Imaging System, Fuji Photo Film Co., Ltd., Minato-ku, Tokyo, Japan.

### 2.2. Synthesis

Para Nitro Tripodal Urea (PNTU): Tris(2-aminoethyl)amine (0.33 g, 2.26 mmol) and *p*-nitrophenyl isocyanate (1 g, 6.09 mmol) were added in dichloromethane (250 mL). The reaction mixture was refluxed for 6 h. A yellowish precipitate was formed and collected by filtration followed by washing with CH_2_Cl_2_ to get desired product. Yield: 1.26 g, 95%. ^1^H NMR (500 MHz, DMSO-*d*_6_, TSP): *δ* 9.32 (*s*, 3H, Ar-N*H*), 8.08 (*d*, *J* = 9.10 Hz, 6H, Ar*H*), 7.58 (*d*, *J* = 8.6 Hz, 6H, Ar*H*), 6.41 (*t*, *J* = 5.75 Hz 3H, CH_2_N*H*), 3.22 (*m*, *J* = 6.15 Hz, 6H, NHC*H*_2_), 2.63 (*t*, *J* = 6.65 Hz, 6H, NC*H*_2_). ^13^C NMR (125 MHz, DMSO-*d*_6_): *δ* 176.64 (*C*=O), 154.8 (Ar-*C*), 147.5 (Ar*C*-NO_2_), 140.8 (Ar-*C*H), 125.4 (Ar-*C*H), 54.1 (NH*C*H_2_), 37.8 (N*C*H_2_). ESI-MS: *m/z* (%) 639.23 [M + H]^+^. Anal. Calcd. for C_27_H_30_N_10_O_6_S_3_: C, 50.78; H, 4.74; N, 21.93. Found: C, 50.77; H, 4.75; N, 21.95.

Penta Fluoro Tripodal Urea (PFTU): A CH_2_Cl_2_ solution (200 mL) of pentafluorophenyl isocyanate (2.62 mL, 20.04 mmol) was added to tris(2-aminoethyl) amine (1 mL, 6.69 mmol) solution (100 mL CH_2_Cl_2_) with constant stirring at room temperature. The reaction mixture was refluxed for 4 h to get a white precipitate, which was collected by filtration. Yield: 4.65 g, 90%. ^1^H NMR (500 MHz, DMSO-*d*_6_, TSP): *δ* 8.32 (*s*, 3H, Ar-N*H*), 6.53 (*t*, *J* = 5.5 Hz, 3H, CH_2_N*H*), 3.15 (*m*, *J* = 6.6 Hz, 6H, NHC*H*_2_), 2.57 (*t*, *J* = 6.65 Hz, 6H, NC*H*_2_). ^13^C NMR (125 MHz, DMSO-*d*_6_): *δ* 154.8 (*C*=O), 144.4 (Ar-*C*), 139.4 (Ar*C*-F), 136.9 (Ar*C*-F), 115.2 (Ar*C*-F), 54.07 (NH*C*H_2_), 38.4 (N*C*H_2_). ESI-MS: *m/z* (%) 774.23 [M + H]^+^. Anal. Calcd. for C_27_H_18_F_15_N_7_O_0_: C, 41.93; H, 2.35; N, 36.84. Found: C, 42.95; H, 2.33; N, 36.86.

Para Nitro Tripodal Thio-Urea (PNTTU): This compound was prepared following the procedure as descried earlier [52].

Penta Fluoro Tripodal Thio-Urea (PFTTU): A solution of pentafluorophenyl isothiocyanate (586.3 µL, 4.15 mmol) dissolved in dichloromethane (50 mL) was added to a solution of tris(2-aminoethyl) amine (200 µL, 1.34 mmol) dissolved in dichloromethane (50 mL) slowly over 0.5 h. The reaction mixture was refluxed for 36 h, yielding a white precipitate. Yield: 0.96 g, 87%. ^1^H NMR (500 MHz, DMSO-*d*_6_, TSP): *δ* 9.30 (*s*, 3H, Ar-N*H*), 8.08 (*s*, 3H, CH_2_N*H*), 3.57 (*t*, *J* = 5.15 Hz, 6H, NHC*H*_2_), 2.73 (*t*, *J* = 6.07 Hz, 6H, NC*H*_2_). ^13^C NMR (125 MHz, DMSO-*d*_6_): *δ* 182.3 (*C*=S), 144.9 (Ar-*C*), 142.9 (Ar*C*-F), 140.5 (Ar*C*-F), 138.2 (Ar*C*-F), 136.2 (Ar*C*-F), 114.9 (Ar*C*-F), 51.9 (NH*C*H_2_), 42.7 (N*C*H_2_). M.P 190 °C. ESI-MS: *m/z* (%) 822.65 [M + H]^+^. Anal. Calcd. for C_27_H_18_F_15_N_7_S_3_: C, 39.47; H, 2.21; N, 11.93. Found: C, 39.45; H, 2.23; N, 11.97.

Thiophene-based Ethylene Macrocyclic Amine (TEA): Diethylenetriamine (1.00 g, 9.70 mmol) and 2,5-thiophenedicarboxaldehyde (1.35 g, 9.70 mmol) were reacted at room temperature under high dilution condensation in MeOH (400 mL), followed by NaBH_4_ reduction. Yield: 1.23 g, 65%. ^1^H NMR (300 MHz, CDCl_3_, TMS): *δ* 6.73 (*s*, 4H, Ar*H*), 3.87 (*s*, 8H, ArC*H*_2_), 3.77 (*t*, 8H, C*H*_2_), 2.72 (*t*, 8H, C*H*_2_) Anal. Calcd. for C_20_H_34_N_6_S_2_: C, 56.8; H, 8.1; N, 19.9. Found: C, 56.6; H, 8.2; N, 20.1.

Copper(II) complex of Methylated Para-xylyl-based Ethylene Macrocyclic Amine (CuMEPEA): The free macrocycle (MEPEA) was prepared by a high dilution condensation reaction of *tera*-phthalaldehyde (0.01 mol) and *N*-methyl-2, 2′-diaminodiethylamine (0.01 mol) in methanol (400 mL) followed by NaBH_4_ reduction. Yield: 1.09 g, 43%. M.p. 88 °C. ^1^H NMR (500 MHz, CDCl_3_, TMS): *δ* 7.19 (*s*, 8H, Ar*H*), 3.75 (*s*, 8H, ArC*H*_2_), 2.78 (*t*, *J* = 5.0 Hz, 8H, NHC*H*_2_), 2.54 (*t*, *J* = 5.0 Hz 8H, NHCH_2_C*H*_2_), 2.16 (*s*, 6H, NC*H*_3_). ^13^C NMR (125 MHz, CDCl_3_): *δ* 138.91 (Ar-*C*), 127.93 (Ar-*C*), 56.50 (CH_3_N*C*H_2_), 53.93 (Ar*C*H_2_), 46.95 (NCH_2_*C*H_2_) 42.14 (N*C*H_3_). ESI-MS: *m/z* (+) 439.3 [M + H]^+^. Anal. Calcd. for C_26_H_42_N_6_: C, 71.19; H, 9.65; N, 19.16. Found: C, 71.33; H, 9.67; N, 19.21. MEPEA (4.0 mmol) dissolved in MeOH (5 mL) and CuBr_2_ (8.0 mmol)dissolved in H_2_O (5 mL) were mixed in MeOH (5 mL). The solution was stirred continuously, and the blue solution was heated to 60 °C for 2 min under stirring. The solution was then kept for crystallization. After ten days, deep blue crystals were collected by filtration, washed by diethyl ether and wiped out with tissue. Yield 167.2 mg, 70% yield, Mp: 214–215 °C (decom). Anal. Calcd. For C_26_H_44_Br_4_Cu_2_N_6_O: C, 34.57; H, 4.91; N, 9.30. Found: C, 34.61; H, 4.90; N, 9.33.

Macrocyclic Amide (MACHAM): The free macrobicycle (1.0 gm, 1.66 mmol) was added to *p*-cyanophenyl isocyanate (1.144 g, 10 mmol) in 250 mL of CH_2_Cl_2_ at room temperature. The reaction mixture was refluxed for 24 h. A white precipitate was formed and collected by filtration, followed by washing with diethyl ether. Yield 90%. ^1^H NMR (500 MHz, DMSO-*d*_6_): *δ* 9.19 (*s*, 6H, CO-N*H*), 7.73 (*d*, 12H, *J* = 14.6 Hz, Ar*H*), *δ* 7.64 (*d*, 12H, *J* = 14.6 Hz, Ar*H*), 7.01 (*s*, 12H, Ar*H*), 4.39 (s, 12H, ArC*H*_2_), 3.16 (*m*, 12H, NC*H*_2_), 1.61 (*m*, 12H, NC*H*_2_). ^13^C NMR (125 MHz, DMSO-*d_6_*): *δ* 155.2 (*C*=O), 146.1 (Ar-*C*N), 138.6 (Ar-*C*), 133.7 (Ar-*C*H), 129.7 (Ar-*C*H), 120.3 (Ar-*C*H), 119.9 (Ar-*C*H), 119.2 (Ar-*C*), 103.8 (Ar-*C*H), 55.7 (N*C*H_2_), 51.7 (N*C*H_2_). ESI-MS: *m/z* 1485.65 [M + Na]^+^. Anal. Calcd. for C_84_H_78_N_20_O_6_: C, 68.93; H, 5.37; N, 19.14. Found: C, 68.95; H, 5.38; N, 19.35.

### 2.3. Cell Culture

The human extravillous CTB cell line Sw.71 utilized in these studies was derived from first trimester chorionic villus tissue and was kindly provided by Gil G. Mor at Yale University School of Medicine, New Haven, CT, USA. These cells are well characterized and share many characteristics with isolated primary cells, including the expression of cytokeratin-7, human leukocyte antigen (HLA) class I antigen, HLA-G, BC-1, CDp, human chorionic gonadotropin, and human placental lactogen [1,2,3,4]. Sw.71 cells were cultured in DMEM/F-12 (Invitrogen) supplemented with 10% fetal bovine serum, 10 mM Hepes, 0.1 mM MEM non-essential amino acids, 1 mM sodium pyruvate, and 100 U/mL penicillin/streptomycin. Cells were incubated at 37 °C, 5% CO_2_, and 99% humidity (Fisher, Isotemp CO_2_ Incubator).

### 2.4. Treatment of Cells

Cells were treated with RPMI Media (Invitrogen) containing 0.1, 1, 10, or 100 nM SR for 48 h. The supernatants were collected to measure the levels of angiogenic and anti-angiogenic factors using ELISA.

### 2.5. ELISA for Angiogenic and Anti-Angiogenic Factors

The supernatants of the TRSPHNO_2_ treated cells were analyzed using a Quantikine ELISA (R&D Systems) for concentrations of sVEGF R1/Flt-1 (sFlt-1), and VEGF. These assays employ the quantitative sandwich enzyme immunoassay technique. Monoclonal antibodies specific for sFlt-1 and VEGF were pre-coated onto the microplate. Standards and samples are pipetted into the wells and any sFlt-1 and VEGF present is bound by the immobilized antibody. After washing away any unbound substances, an enzyme-linked polyclonal antibody specific for either sFlt-1 and VEGF is added to the wells. Following a wash to remove any unbound antibody-enzyme reagent, a substrate solution is added to the wells, and color develops in proportion to the amount of sFlt-1 and VEGF bound in the initial step. The color development is stopped and the intensity of the color is measured using a plate reader (SPECTRAmax 340PC384).

### 2.6. Western Blots for VEGFR-1, AT_1_, and AT_2_ Receptors

Cells were treated with RPMI Media (Invitrogen) containing 0.1, 1, 10, or 100 nM SR for 48 h. The cell lysates were utilized to measure VEGFR-1, AT_1_, and AT_2_ receptor expression. Protein concentrations of cell lysates were determined using a BCA Protein Assay Kit (Pierce). An equal amount of protein from each sample was run on a NuPAGENovex 4–12% Bis-Tris Gel (Invitrogen) and transferred to 0.45 μm nitrocellulose membrane (Bio-Rad). Membranes were blocked in 5% milk and incubated with VEGFR-1 (Invitrogen), AT_1_, (Santa Cruz Biotechnology), and AT_2_ (Abcam) antibodies. After the addition of the corresponding secondary antibody (Jackson ImmunoResearch Laboratories), proteins were visualized with SuperSignal West Dura Chemiluminescent Substrate (Pierce) and a chemiluminescence detection system (LAS-3000 Imaging System). Densitometry was measured using ImageJ software (National Institutes of Health, Bethesda, MA, USA,) and normalized using β-actin (ABCAM, Cambridge, MA, USA).

### 2.7. Statistical Method

Data are presented as mean ± SEM. Data from CINO-treated groups were compared to basal (DMSO)-treated groups using a one-way analysis of variance with Tukey’s post hoc test. A *p*-value of less than 0.05 was considered significant.

## 3. Results

### 3.1. PNTU, PFTU, PNTTU, PFTTU, and CuMEPEA Downregulated Angiogenic Factors

**Figure 3:**
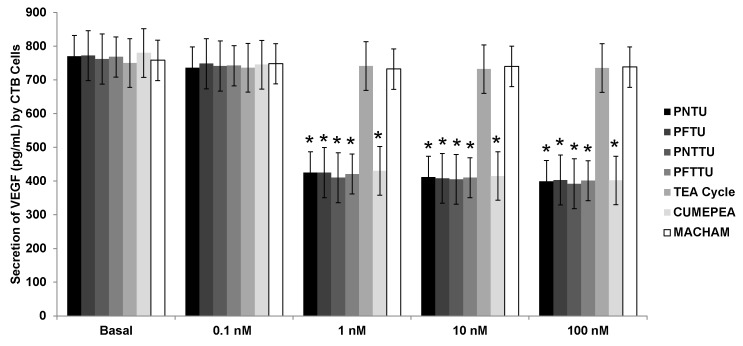
Cytotrophoblast (CTB) cells were treated with different concentrations of synthetic receptors (SRs), and the levels of Vascular Endothelial Growth Factor (VEGF) were measured in the cell culture media by Enzyme-Linked Immunosorbent Assay (ELISA). The SRs significantly (* *p* < 0.05) downregulated the secretion of VEGF by CTB cells. The results are presented as the mean ± Standard Error Mean (SEM) (n = 6, 4 replicates each).

The secretion of VEGF was inhibited in the culture media of CTB cells treated with ≥1 nM of PNTU, PFTU, PNTTU, PFTTU, and CuMEPEA while the angiogenic factors in the culture media of CTB cells had no effect when they were treated with 0.1 nM of those compounds. TEA Cycle and MACHAM had no effect on VEGF secretion in CTB cells (Figure 3). These results suggested that the SR had an anti-angiogenic effect on CTB cells environment.

### 3.2. PNTU, PFTU, PNTTU, PFTTU Upregulated Anti-Angiogenic Factors

**Figure 4:**
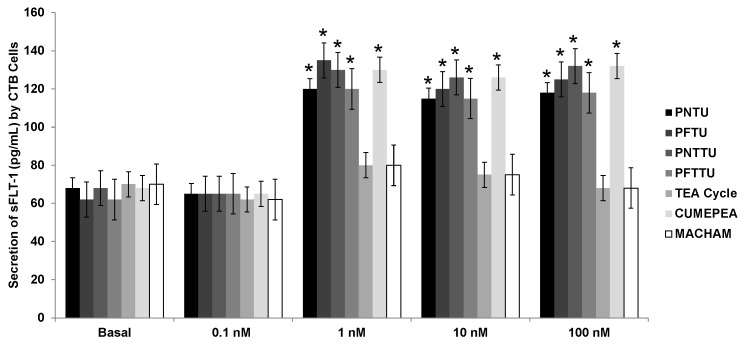
CTB cells were treated with different concentrations of SRs, and the levels of sFlt-1 were measured in the cell culture media by ELISA. The SRs significantly (* *p* < 0.05) upregulated the secretion of sFlt-1 by CTB cells. The results are presented as the mean ± SEM (n = 6, 4 replicates each).

The secretion of sFlt-1 was significantly upregulated in the culture media of CTB cells treated with ≥1 nM PNTU, PFTU, PNTTU, and PFTTU, while the secretion of sFlt-1 did not have much effect on the culture media of CTB cells treated with 0.1 nM of PNTU, PFTU, PNTTU, and PFTTU. TEA Cycle and MACHAM had no effect on sFlt-1 secretion. (Figure 4) Therefore, the SRs have demonstrated an anti-angiogenic effect on the CTB cells.

### 3.3. PNTU, PFTU, PNTTU, PFTTU, CuMEPEA, TEA Cycle and MACHAM Upregulated AT_2_ Receptor Expression

**Figure 5:**
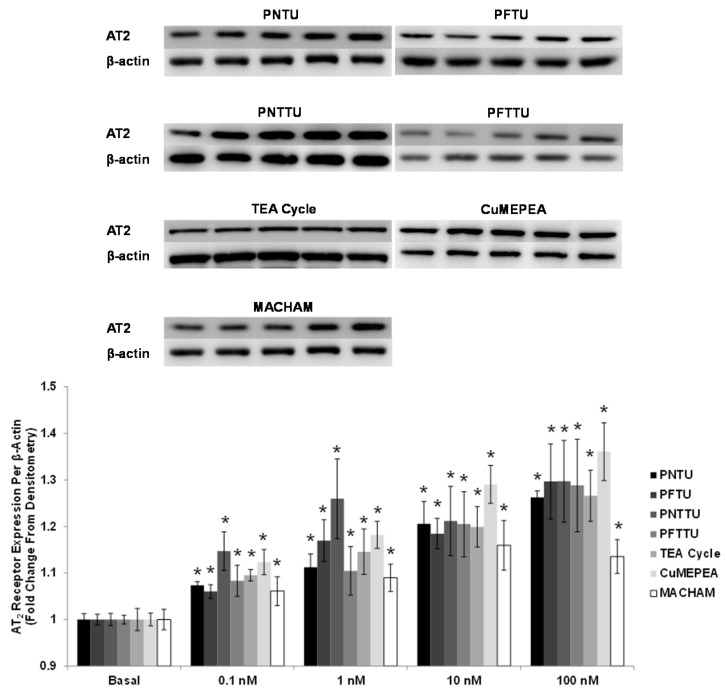
CTB cells were treated with different concentrations of SRs, and Angiotensin II receptor, type 2 (AT_2_) receptor expression was measured in the cell lysates by Western Blot. The SRs significantly (* *p* < 0.05) upregulated the expression of AT_2_ receptor in CTB cells. The data are presented as mean ± SEM for four experiments. One blot from each of the seven SRs is shown.

The AT_2_ receptor expression was significantly upregulated in the culture media of CTB cells treated with ≥1 nM PNTU, PFTU, PNTTU, PFTTU, CuMEPEA, TEA cycle, and MACHAM compared to basal (Figure 5). There is a possibility that AT_2_ receptor mediated vasodilation plays a role in modulating Ang-II contractile responses in pregnancy.

### 3.4. PNTU, PFTU, PNTTU, PFTTU, CuMEPEA, TEA and MACHAM Downregulated VEGFR-1 and AT1 Receptor Expression

**Figure 6:**
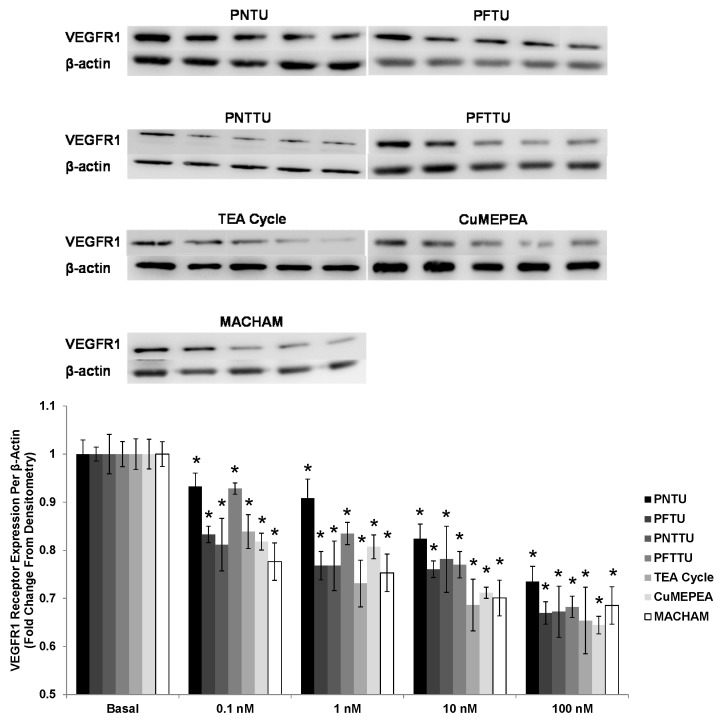
CTB cells were treated with different concentrations of SRs, and Vascular endothelial growth factor receptor 1 (VEGFR1) receptor expression was measured in the cell lysates by Western Blot. The SRs significantly (* *p* < 0.05) downregulated the expression of VEGFR1 receptor in CTB cells. The data are presented as mean ± SEM for four experiments. One blot from each of the seven SRs is shown.

The VEGFR-1 and AT1 receptor expression was downregulated in the culture media of CTB cells treated with ≥1 nM PNTU, PFTU, PNTTU, PFTTU, CuMEPEA, TEA cycle, and MACHAM, compared to basal (Figure 6). However studies have demonstrated that the activation of AT_1_ receptor appears to mediate hypertension associated with excessive IL-6 during preE.

### 3.5. PNTU, PFTU, PNTTU, PFTTU, and CuMEPEA Downregulated AT_1_

**Figure 7:**
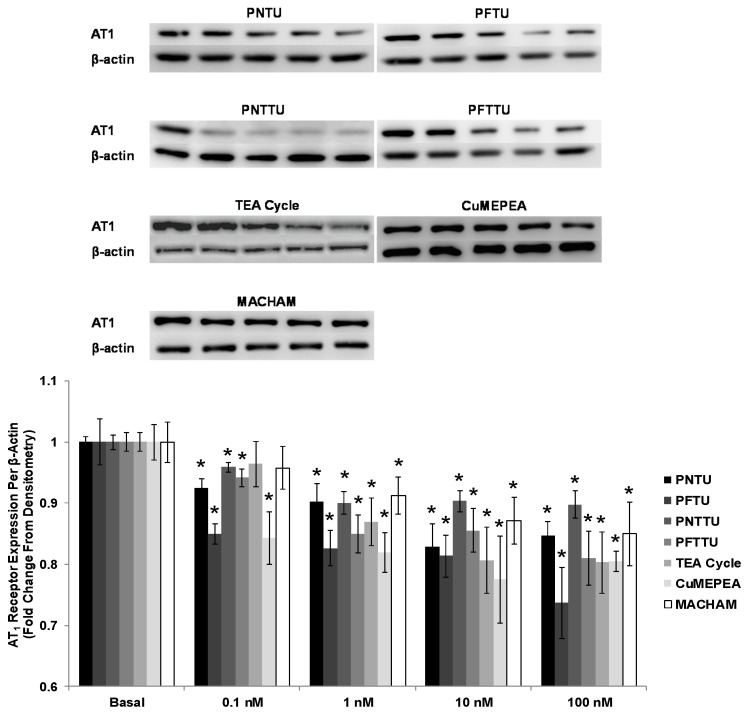
CTB cells were treated with different concentrations of SRs, and Angiotensin II receptor, type 1 (AT_1_) receptor expression was measured in the cell lysates by Western Blot. The SRs significantly (* *p* < 0.05) downregulated the expression of AT_1_ receptor in CTB cells. The data are presented as mean ± SEM for four experiments. One blot from each of the seven SRs is shown.

The expression of AT_1_ receptor expression was significantly downregulated for CTB cells treated with 0.1 and 1 nM PNTU, PFTU, PNTTU, PFTTU, and CuMEPEA; however AT_1_ receptor expression was not changed in CTB cells treated with 10 and 100 nM of those compounds (Figure 7).

## 4. Discussion

As compared to preE pregnancies, normal pregnancies reveal a higher ratio of angiogenic factors to anti-angiogenic factors that then ensures the human extra villous CTB cells to proliferate, migrate, and invade the chorionic villus tissue for the establishment and growth of a healthy placenta. Two angiogenic factors that are vital for normal placental progression are VEGF and PlGF. Within an individual who has developed preE, the anti-angiogenic factors are produced at elevated levels. Through varying data and tests that we have run, such as the ELISA and the Western Blot [27], we propose that cardiotonic steroids being secreted throughout the body may cause this imbalance. The CTS inhibited CTB proliferation, migration, invasion, and ERK1/2 phosphorylation, and they activated Jnk1/2 phosphorylation, p38 phosphorylation, and apoptosis, as evaluated by caspase 3/7 and annexin-five staining [27,28]. The CTS also arrested cell cycle progression without causing a cytotoxic effect on the cells [27,28]. We have demonstrated that CTS-induced impairment of CTB cell function occurs via the modulation of MAPK signaling, cell cycle arrest, and the activation of apoptosis. However, the cell surface receptors for CTS have been poorly understood. CINO impairs CTB cell function via cell cycle arrest and apoptotic signaling [32]. Since mechanistic insights of the release of the cardiotonic steroids are still not well known, the proper selection of effective functional groups of synthetic compounds may elucidate the location of cardiotonic binding to the CTB cells. This can be accomplished by the binding of multi-functional SRs to MBG, which enables the tracking of MBG to the binding sites. In the long run, this may help us find a treatment to help prohibit the premature apoptosis signaling in CTB cells. In order to better understand the cell surface receptors for CTS, we decided to test seven selected new compounds; namely, (PNTU), (PFTU), (PNTTU), (PFTTU), TEA, CuMEPEA, and MACHAM. When looking at the CTB cells treated with ≥1 nM of PNTU, PFTU, PNTTU, PFTTU, and CuMEPEA, the secretions of VEGF and PlGF were decreased. However, TEA and MACHAM had no effect on VEGF secretion. When observing anti-angiogenic regulation, the secretion of sFlt-1 and sEnd were increased in the CTB cells treated with ≥1 nM PNTU, PFTU, PNTTU, PFTTU, and CuMEPEA. However, TEA and MACHAM had no effect on sFlt-1 secretion. It is proposed that the urea/thiourea [HN(C=O/S) NH] groups in PNTU, PFTU, PNTTU, and PFTTU possibly interact with the active sites of cells through hydrogen bonding interactions. In addition, the presence of three electron-withdrawing substituents (as *p*-nitro groups or penta fluoro groups) enhances the acidity of the attached NH groups, thereby increasing the overall activity of the ligand, as well as the conformational flexibility with six H-donor groups, which may allow the ligand to interact with cells more comfortably [51]. The receptor CuMEPEA also interacts with the cells, possibly due to the presence of the active Lewis acid centers as copper(II) within the receptor’s framework. In contrast, TEA and MACHAM, which contain different functional groups, amines, and amides, respectively, exhibited poor interactions with the cells under the experimental conditions. These observations suggest that the poor acidity of the two functional groups diminishes the ability of these compounds to interact with the cells under neutral conditions [43,44]. The AT_2_ receptor expression was significantly upregulated; however, the AT_1_ and VEGFR-1 receptor expression was significantly downregulated in CTB cells treated with with ≥1 nM PNTU, PFTU, PNTTU, PFTTU, and CuMEPEA. However, TEA and MACHAM showed negligible interactions due to the presence of amine and amide functionalities, which are less acidic than urea and thiourea functionalities under neutral conditions [44]. AT_2_ receptor is antiangiogenic and antiproliferative; however, AT_1_ and VEGFR1 receptors are angiogenic [54]. AT_1_ and AT_2_ have discrete physiological actions from each other; activation of AT_1_ induces vasoconstriction and cell proliferation, while AT_2_ facilitates vasodilatation, hinders cell growth and triggers apoptosis [55]. Thus these data confirm our data on VEGF and s-Flt-1 as well as our previous data for CTS and SR [28,29,33,52,56].

PreE is a hypertensive disorder that develops in three to 10 percent of pregnant women during their first trimester of gestation [57,58]. In the first trimester of pregnancy, the CTB cells of the extra villous trophoblast column migrate and invade the decidualized endometrium attaching the placenta to the uterus. The CTB cells subsequently breach and line the uterine blood vessels, leading to the channeling of the maternal blood to the placenta. Adequate CTB invasion leads to the remodeling of the maternal vessels, which is essential for the fetus, which needs increased maternal blood flow as the pregnancy progresses. Defects in CTB differentiation are often associated with preE since there will be a reduction in the uteroplacental perfusion that will further lead to placental focal ischemia and hypoxia later in pregnancy.

In addition to inhibiting CTB proliferation, migration, and invasion, MBG has been shown to increase phosphorylated p38 concentration in CTBs, which is considered to be the active protein form that initiates a stress signaling cascade [27,28]. It has been shown that hypoxic stress activates p38 signaling in villous trophoblasts in preE patients [37]. COX-2, a prostaglandin endoperoxide synthase, catalyzes the conversion of prostaglandin from arachidonic acid and is considered to be at an increased level in the early trophoblast tissue [35,36,37]. It has been shown that COX-2 is increased in the placenta tissue of preE patients [38,39]. However, the exact mechanism by which COX-2 is increased in preE and the interaction between CTSs and COX-2 is not well understood.

## 5. Conclusions

During this study, we have explored several synthetic receptors by varying the functional groups, including ureas, thioureas, metal centers, amines, and amides to identify their effectiveness on an angiogenic profile of CTB cells. In particular, we have shown that the receptors containing urea/thiourea groups are highly effective at downregulating CTB proliferation without any cytotoxic effect on the CTB cells. This has been proven by a cell viability test. Our results demonstrated that the synthetic receptors significantly decreased CTB cell proliferation at 100 nM, whereas cell viability at 100 nM remains the same as at basal and lower concentrations [33,34,56]. The molecules containing active binding sites and defined cavities are known to provide strong affinity for anionic species through H-bonding and electrostatic interactions under neutral conditions [44]. We propose that the specific binding sites within the synthetic receptors possibly interact with membrane receptors through residual anionic carboxyl groups, resulting in the decrease in signaling for an Angiogenic profile. The results obtained from this study will be useful to design an effective inhibitor to prevent the CTS-induced impairment of CTBs. Taken together, the results from our study will serve as a prototype for the rational design of highly efficient synthetic receptors for biomedical applications.
